# Detection rate of dental trauma and maxillofacial injuries in high-energy polytrauma patients on total body CT: incidence and underestimation

**DOI:** 10.1007/s10140-025-02376-9

**Published:** 2025-08-23

**Authors:** Nicola Maria Lucarelli, Fabio Panarelli, Alessia Spitaleri, Carlotta Testini, Chiara Morelli, Giovanni Lorusso, Ilaria Villanova, Sara Greco, Nicola Maggialetti

**Affiliations:** Interdisciplinary Department of Medicine, Section of Radiology and Radiation Oncology, University of Bari”Aldo Moro”, Bari, 70124 Italy

**Keywords:** Emergency unit, Total body CT, Maxillofacial injuries, Incidental findings

## Abstract

**Purpose:**

The aim of our study was to assess the frequency and detection rate of dental trauma and maxillofacial injuries (DTMI) in high-energy polytrauma patients undergoing total-body computed tomography (TBCT) and to estimate how often they are correctly reported by the radiologists.

**Methods:**

This retrospective study included 611 patients who underwent TBCT following high-energy trauma between July 2024 and February 2025. DTMI findings were analyzed based on initial radiology reports and retrospective image review.

**Results:**

DTMI was identified in 124 of 611 patients (20.2%), of which only 76 cases (61%) were reported at admission. Dental trauma was significantly underreported (76% missed rate), with only 12 of 50 cases (24%) initially recognized. In contrast, maxillofacial injuries were detected in 75 of 97 cases (77,3%). Most DTMI-positive patients (82/124, 66%) had additional traumatic findings, including cranial or spinal injuries (33/124, 26.6%) and multi-district trauma (29/124, 23.4%). Only 20 patients (16%) presented with isolated facial trauma. A small subgroup (18/124, 14.5%) had DTMI with no other traumatic findings, suggesting possible oversight in the absence of overt injury. Underreporting rates were slightly higher during night shifts (55%) compared to daytime (48%), though not statistically significant (χ² = 0.654, *p* > 0.05). DTMI was more common in males (ratio 1.79:1).

**Conclusion:**

DTMI, especially dental trauma, is frequently underdiagnosed in the acute trauma setting. Improved detection may be achieved through dedicated imaging protocols, increased radiologists awareness, and AI-based support tools.

**Graphical abstract:**

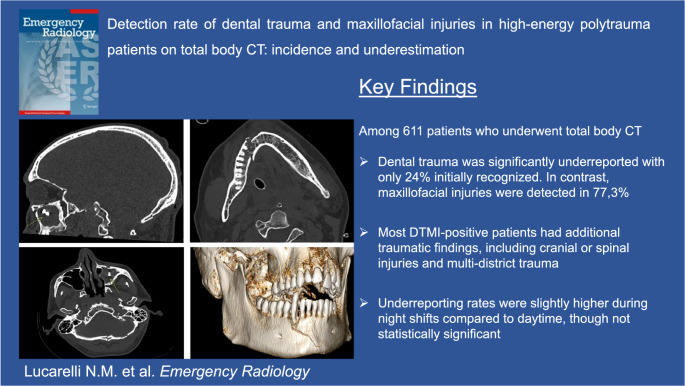

## Introduction

Polytrauma is often associated with cranio-encephalic and maxillofacial injuries, where accurate diagnostic evaluations and timely treatments significantly influence patient outcomes, allowing functional recovery and reducing complications [1, 2].

Maxillofacial injuries affect 15% of severely traumatized patients, with 71% of cases involving the upper two third of the face while mandibular fractures isolated or associated with dental injuries represent 24%. Dental injury is one of the most common findings encountered during facial trauma, with an estimated incidence of 13.1% in polytraumatized patients [3].

In the emergency setting, primary assessment is aimed at determining the hemodynamic stability of the polytrauma patient. This is followed by comprehensive imaging, in which computed tomography (CT) plays a key role as the gold standard for rapid and systematic evaluation during the so-called “golden hour” [4], allowing assessment of trauma severity and detection of life-threatening injuries such as parenchymal damage and vascular involvement. While CT has demonstrated high sensitivity and specificity for many critical traumatic conditions (up to 75% and 99.1%, respectively, for penetrating thoracic and mediastinal injuries) [5], these values do not directly apply to dental and maxillofacial injuries, whose detection may be lower and depends on image quality, radiologist expertise and trauma protocols [6].

Radiologists tend to give priority to potentially lethal injuries and other major incidental findings, resulting in underestimation of less urgent but significant traumas such as dental trauma and maxillofacial injuries (DTMI). These injuries can have a significant long-term impact on the morbidity and overall quality of life of the patient, especially in the presence of airway compromise, functional and aesthetic deficits or soft tissue lacerations [7, 8].

The aim of our study was to assess the frequency and detection rate of dental trauma and maxillofacial injuries (DTMI) in high-energy polytrauma patients undergoing total-body computed tomography (TBCT) and to estimate how often they are correctly reported by the radiologists.

Failure to promptly detect dental trauma and maxillofacial fractures in polytrauma patients can lead to serious clinical consequences, including airway compromise, persistent pain, infection, malocclusion, functional deficits, and aesthetic deformities—all of which negatively affect recovery and quality of life. For instance, as reported in the literature by Sasaki et al., missed mandibular fractures can lead to airway obstruction requiring emergency intubation and surgical intervention [9]. Similarly, Jain et al. demonstrated that delayed diagnosis of maxillofacial fractures was associated with higher rates of infection, prolonged hospitalization, and increased need for reconstructive surgery [10].

## Materials and methods

### Study population

This study was conducted in accordance with ethical principles and in compliance with the applicable legal and regulatory requirements under Italian law.

A retrospective observational cohort study was performed on 611 patients (392 male, 219 female) who underwent total body CT scans following high-energy trauma and presented to our emergency department between July 2024 and February 2025. The inclusion criteria were: (a) patients involved in high energy politrauma (b) patients who underwent whole body CT (c) unconscious patients in whom high-energy polytrauma was strongly suspected based on their critical clinical condition and comprehensive physical examination. The exclusion criteria were: (a) patients with non-removable dental prosthesis that caused artifacts compromising image interpretation (b) patients with direct head trauma (c) patients with poor-quality CT scans that did not allow for adequate evaluation of the DTMI evaluation.

### Classification of maxillofacial and dental trauma

**Fig. 1:**
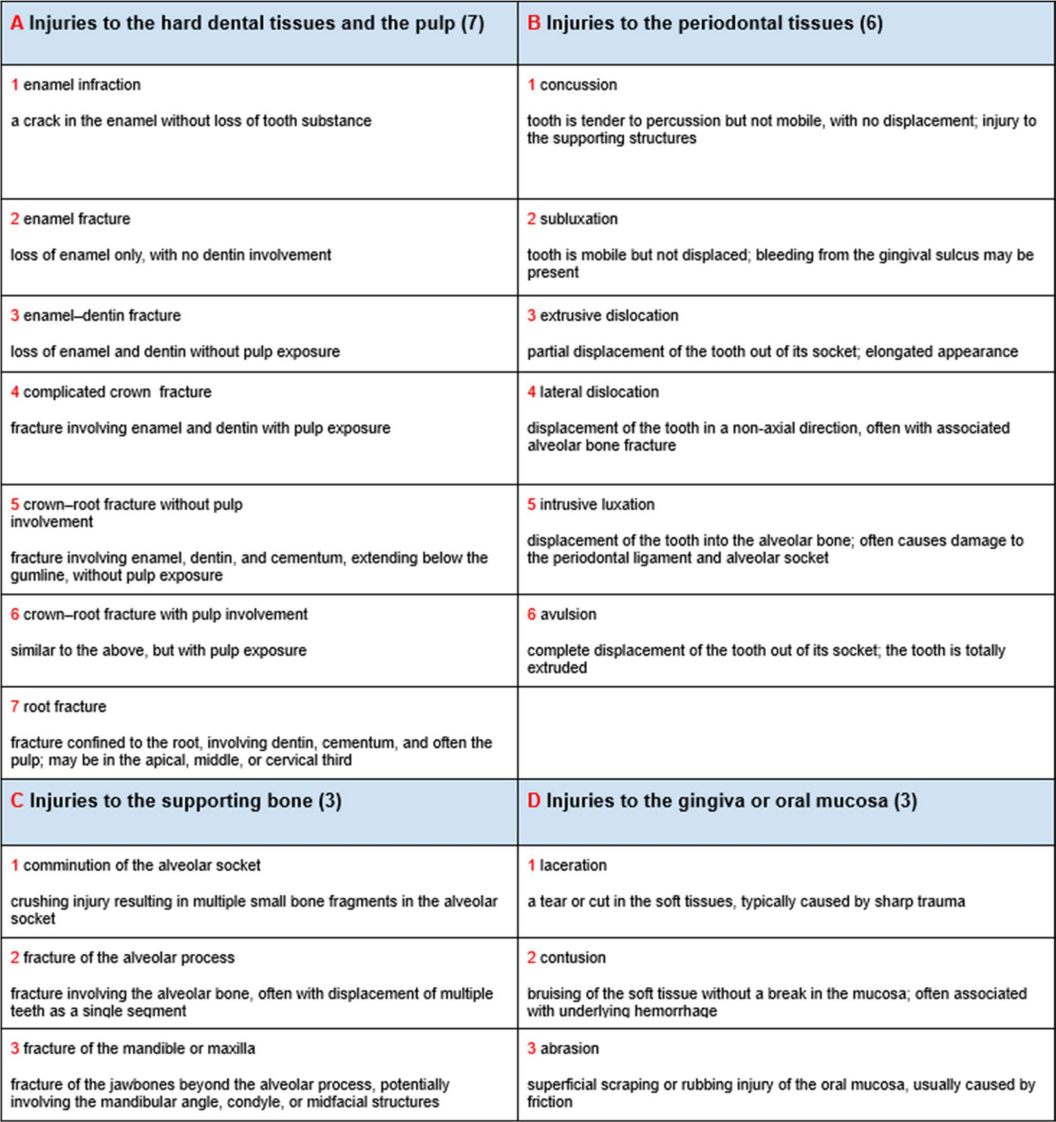
Andreasen classification of traumatic dental injuries − 19 Categories [1]:

In this study, dental injuries were classified according to the Andreasen classification (Fig. 1), which provides a comprehensive and objective framework encompassing 19 injury categories involving teeth, supporting structures, gingiva, and oral mucosa. This system distinguishes between various forms of tooth displacement, including intrusive, extrusive, and lateral luxations, thus minimizing interpretative variability [1].

**Fig. 2:**
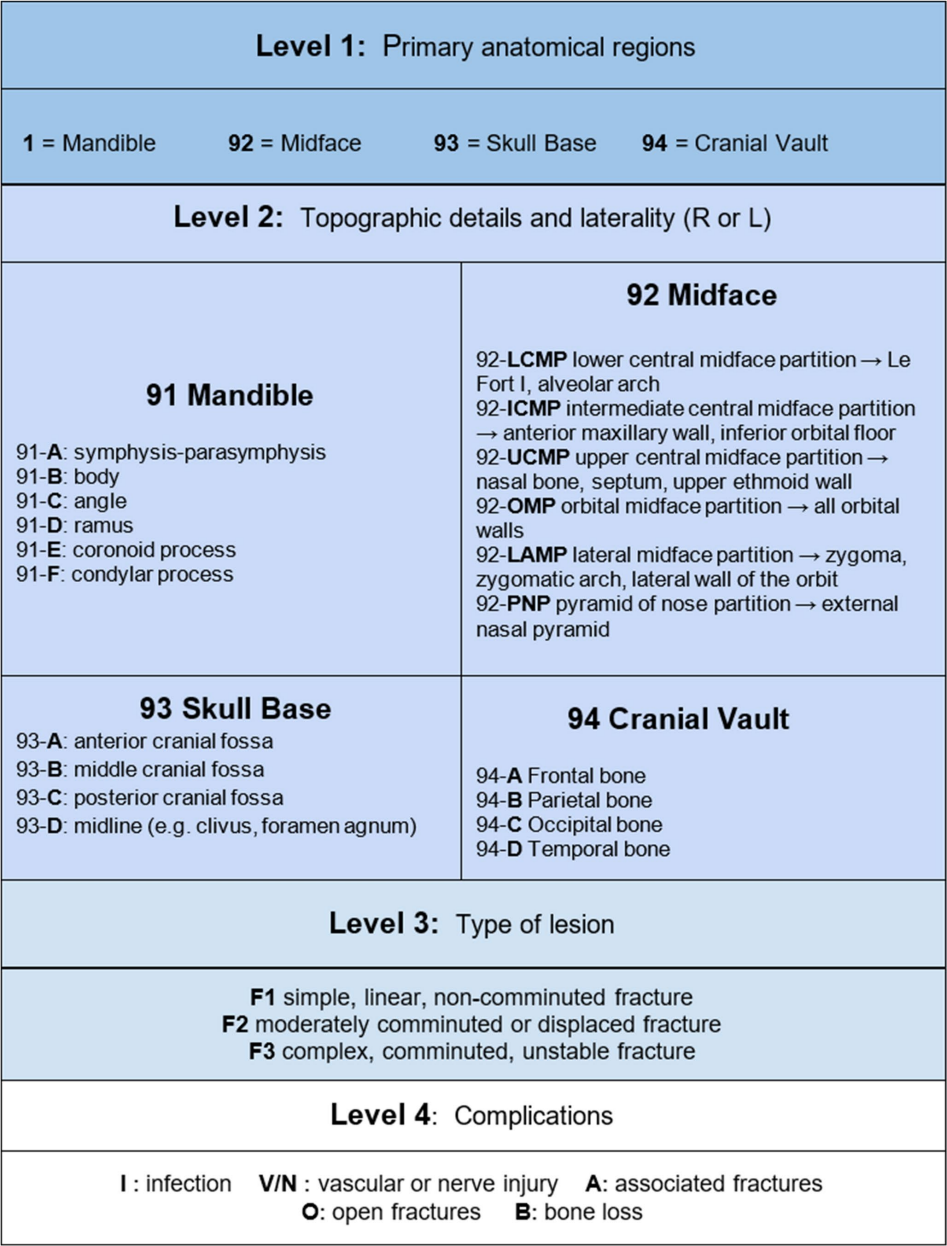
AO-CMF classification of traumatic maxillofacial injuries [11]

Maxillofacial fractures were categorized using the AO-CMF classification system, developed by the AO Foundation [11] (Fig. 2). This system offers a detailed, anatomically accurate, and radiologically integrated classification scheme. It divides the craniofacial skeleton into four main regions—the mandible, midface, cranial base, and cranial vault—and further into subunits, using a standardized alphanumeric coding system. This approach enhances reproducibility and consistency in reporting, facilitating both clinical decision-making and multicenter data sharing.

### Scan protocol

All CT examinations were performed using a 128 slice multidetector CT scanner (Siemens Somatom Definition DS). An unenhanced scan was first acquired in the supine position, extending from the head to the pubic symphysis, followed by contrast enhanced multiphase scans in accordance with the European Society of Emergency Radiology’s guidelines on radiological polytrauma imaging [12]. CT was performed through the injection of 1 ml/kg of iodinated contrast agent followed by 40 ml of saline solution at a flow rate of 3.5 ml/s, into a peripheral vein. A bolus-tracking technique was used, with the region of interest placed in the supraceliac abdominal aorta and a trigger threshold set at 100 Hounsfield Units (HU).The scan delay was 6s. Images were acquired in free breath using the following parameters: 0.6 mm slice thickness, 100 kVp tube voltage, 0.33 s rotation time, pitch of 1.2, and a total acquisition time 2.94 s. In addition to standard soft-tissue reconstructions, high-resolution images (1 mm slice thickness) were reconstructed using a sharp bone algorithm (J70 kernel), as part of our institutional polytrauma protocol, and were systematically reviewed for detailed assessment of facial bones and skull base structures. Reconstruction images with a 1 mm slice thickness were then archived using our institutional PACS (Enterprise Imaging, Biesse Medica, Roma, Italy).

### Report analysis

Emergency room records were retrospectively reviewed by three radiologists with different years of experience in trauma imaging, to identify cases of high energy polytrauma and to assess whether the corresponding CT reports adequately documented DTMI [1]. The radiologists initially evaluated the CT images blinded to both clinical information and original reports, to minimize interpretation bias. Subsequently, the original reports were reviewed to calculate the detection rate. The images were evaluated using 3-dimensional reconstructions. To optimize the detection of osseous and dental injuries, bone window settings were standardized with a window width (WW) of approximately 2500 Hounsfield Units (HU) and a window level (WL) near 500 HU. Reconstructions were performed using a high-resolution sharp bone convolution kernel (J70), which enhances edge definition and detail of bony structures.

Reconstruction images with a 1 mm slice thickness were archived in our institutional PACS system (Enterprise Imaging, Biesse Medica, Rome, Italy). Image evaluation was conducted using 3D volume-rendered reconstructions, supplemented by multiplanar reformats (MPR) as needed to provide comprehensive anatomical visualization. The MPR and 3D volume-rendered reconstructions were not systematically reviewed during the initial emergency reporting, as the primary CT interpretation in our institution follows a time-sensitive, total-body trauma protocol primarily focused on life-threatening injuries. These advanced reconstructions were instead retrospectively reviewed during the study analysis phase to optimize the detection of DTMI.

Any discrepancies between readers were resolved by consensus through open discussion.

### Statistical analysis

Statistical analysis was performed using IBM SPSS software (version 30.0, SPSS Inc., Armonk, NY, USA). Descriptive statistics, including means, standard deviations (SD) and percentages, were used to summarize the data.

Chi-square test were applied to assess differences between observed and expected frequencies. Where appropriate, pairwise comparisons between subgroups were conducted using 2 × 2 Chi-square tests. A p-value < 0.05 was considered statistically significant.

Cramér’s V was calculated to assess the strenght of association between categorical variables, interpreted as weak (V < 0.1), moderate (0.1 ≤ V < 0.3), or strong (V ≥ 0.5). Odds ratios (ORs) with 95% confidence intervals (CIs) were calculated to estimate effect sizes.

## Results

A total of 611 patients were retrospectively reviewed. Among them, 392 were male (64%) and 219 were female (36%), with a male-to-female ratio of 1.79:1. The mean patient age was 57.5 years (SD ± 23.4), with cases approximately equally distributed across all age groups [13–15].

The overall incidence of DTMI was 20.2% (124 out of 611 patients). Of these, 76 patients (61%) were correctly identified in the initial CT reports, while 48 patients (39%) were detected only during retrospective review. Among the 124 patients with DTMI, a total of 147 traumatic findings were recorded, comprising 97 (78,2%) facial bone injuries and 50 (40,3%) dental injuries. Lesion-based analysis revealed an overall detection of 59% (87 out of 147 lesions). When analyzed separately, the detection rate for maxillofacial injuries was 77% (75 out of 97), whereas dental trauma was detected in only 24% of cases (12 out of 50).

**Fig. 3:**
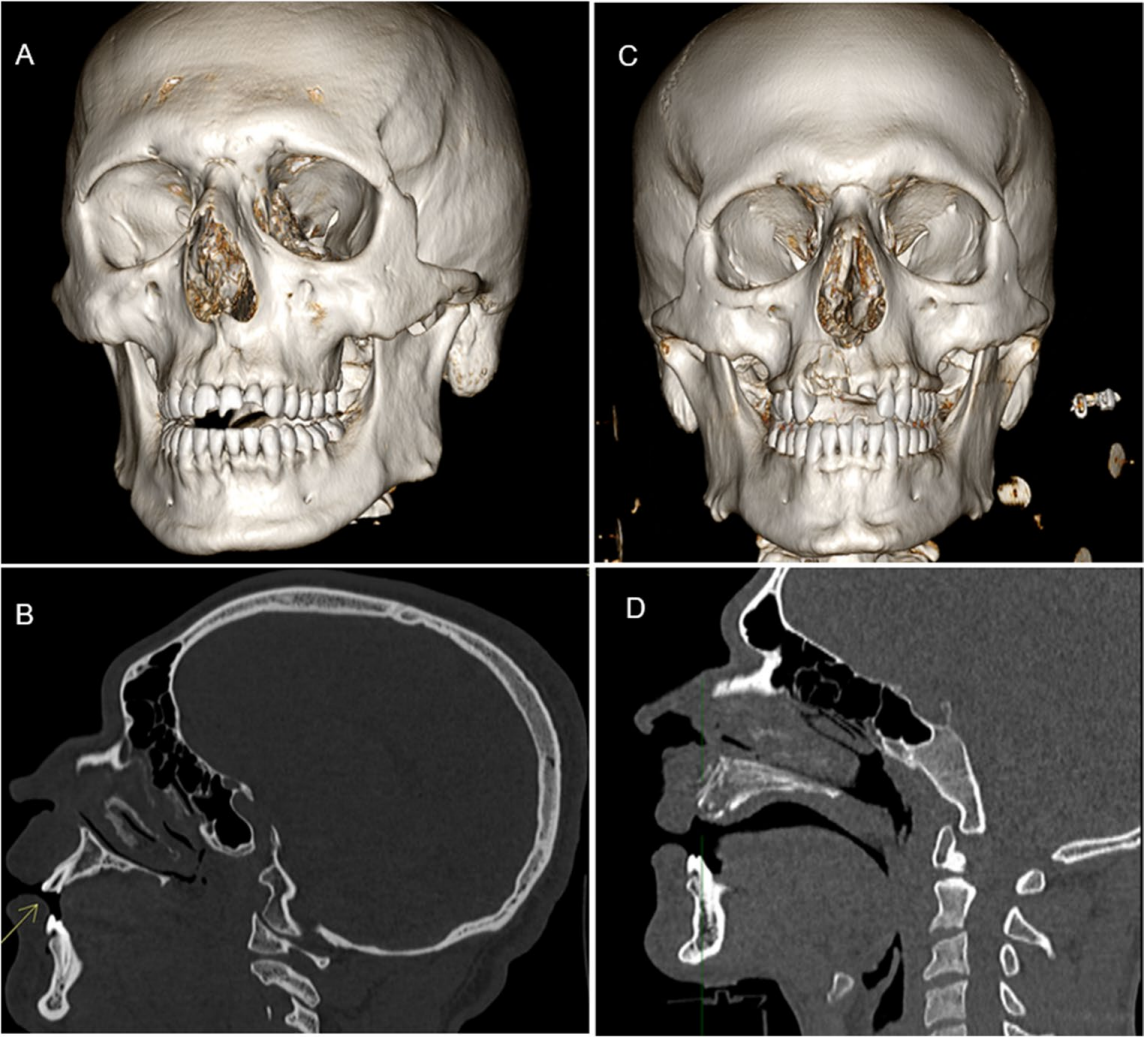
**A**, **B**: crown fracture of 2.1, 2.2 (A3 in Andreasen classification). **C**, **D**: Fracture extending from tooth 1.1 to 1.4, involving the nasal bones and the right maxilla (A6B6 in Andreasen classification)

**Fig. 4:**
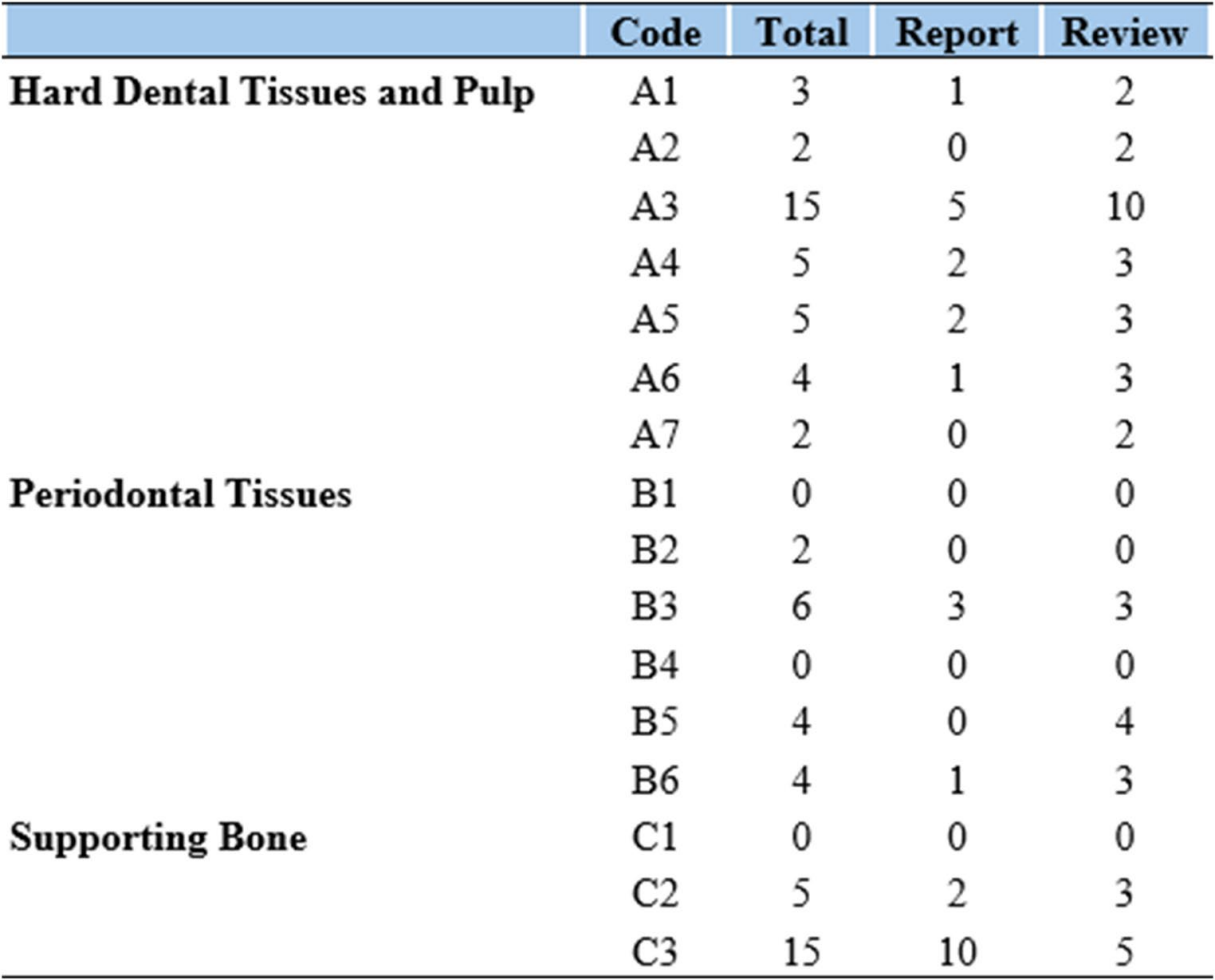
Dental lesions found according to Andresen [1]:

The most frequently detected type of dental trauma was enamel-dentin fracture (A3), found in 15 of 50 cases (30%), followed by alveolar process fractures and crown-root fractures with pulpal involvement, each observed in 5 cases (10%) (Fig. 3). Among periodontal injuries, the most common lesion was extrusive dislocation (B3), observed in 6 cases (12%). Supporting bone injuries (C3) represented the most frequent form of osseous trauma, accounting for 15 of 50 cases (30%) (Fig. 4).

**Fig. 5:**
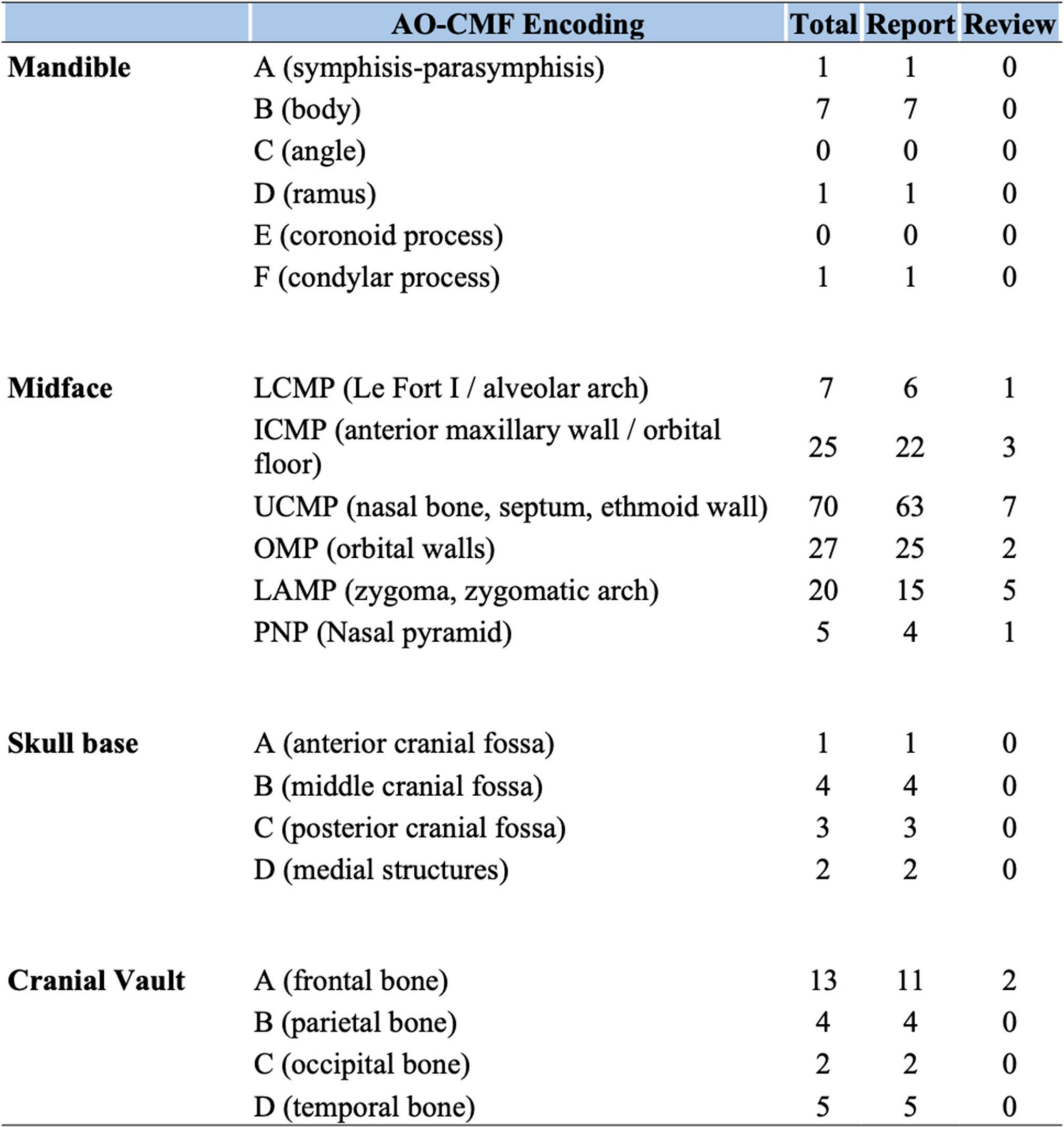
Maxillofacial lesions found according to AO-CMF* 11 :

**Fig. 6:**
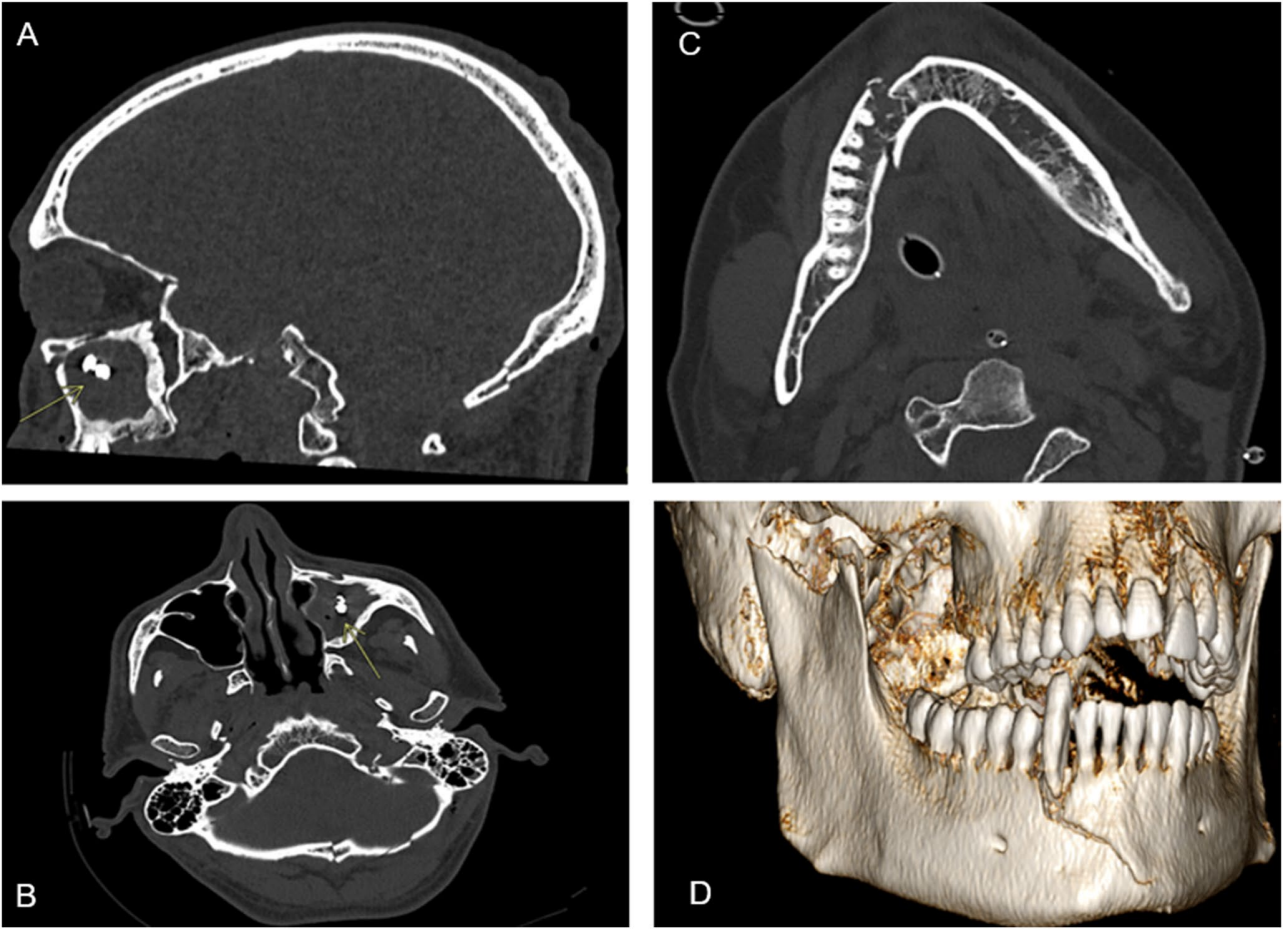
**A**, **B**: Displaced tooth within the maxillary sinus (B6 in Andreasen classification, 94 C in AO-CMF classification); **C**,**D**: Right condyle and parasymphysis fracture with concomitant dental avulsion (B6C3 in Andreasen classification, 91 A,91 F in AO-CMF classification)

Facial bone involvement was predominantly localized in the midface region. Upper central midface partition (UCMP), involving the nasal bone, septum and upper ethmoid wall, were observed in 70 patients (72%), followed by orbital midface partition (OMP), involving all orbital walls, observed in 27 patients (28%) and intermediate central midface partition (ICMP), involving anterior maxillary wall and inferior orbital floor observed in 25 patients (26%) (Figs. 5 and 6).

A Chi-square goodness-of-fit test revealed a statistically significant deviation in the distribution of dental trauma types (χ² = 68.00, df = 16, *p* < 0.0001). Further pairwise Chi-square analyses showed that enamel-dentin fractures (A3) and supporting bone fractures (C3) were significantly more frequent than other trauma types (χ² = 24.41, df = 1, *p* = 0.0001). Similarly, grouped midfacial fractures types (UCMP, OMP, ICMP) showed a significantly overrepresentation (χ² = 10.69, df = 1, *p* = 0.0011).

Among the 124 patients with DTMI, 25 had no other associated lesions; 33 had neuroradiologically relevant associated injuries (15 involving the brain, 8 the spine, and 10 both brain and spine); 20 had body injuries requiring general radiological or interventional evaluation; 29 had combined neuro-body injuries; and 18 were considered completely negative for other traumatic findings at the initial CT.

A statistically significant and strong association was found between the presence of associated injuries (e.g., brain, spine, body) and positive maxillofacial findings at admission CT (χ² ≈ 85; *p* < 0.0001; Cramér’s V = 0.82). This finding suggests that maxillofacial injuries are more likely to be detected when associated with other severe injuries, while isolated cases are at greater risk of being overlooked.

Regarding the timing of admission, 81 out of 124 trauma patients (65,3%) presented during daytime hours. Among these, 42 cases (51,9%) were identified at admission, while 39 cases (48,1%) were identified only during retrospective review. In contrast 43 out of 124 trauma patients (34,7%) occurred during the nighttime hours, with 19 cases (44,2%) identified at admission and 24 cases (55,8%) identified retrospectively. The association between shift time (day vs. night) and detection timing was weak and not statistically significant (Cramér’s V = 0.073; OR = 1.36; χ² = 0.654; *p* > 0.05), suggesting a non-significant trend toward slightly improved detection during daytime.

### Associated findings

The analysis revealed the presence of multiple concomitant traumatic injuries, suggesting a potential correlation with DTMI.

Notably, a statistically significant association was observed between DTMI and traumatic brain injuries, particularly intracranial hemorrhages, including intraparenchymal, subarachnoid, and subdural bleeding.

Vertebral dislocations are frequently observed in high-energy polytrauma and contribute to complex clinical presentations in which maxillofacial and dental injuries may be initially underestimated or diagnosed late. Concomitant thoracoabdominal injuries—such as pulmonary, hepatic, and splenic contusions, as well as pneumothorax, pneumomediastinum, hemoperitoneum, and hemothorax—are commonly reported, often secondary to rib and pelvic fractures or severe visceral trauma. These associated lesions reflect the transmission of substantial kinetic energy throughout the body, increasing the risk of maxillofacial involvement and complicating both diagnosis and timely management of facial and dental injuries.

## Discussion

This retrospective analysis revealed that 124 of 611 patients (20.2%) admitted for high-energy polytrauma had DTMI. However, only 76 of these patients (61.3%) had such injuries adequately described in the initial radiological reports. An additional 48 cases (38.7%) were identified retrospectively, resulting in an overall DTMI detection rate of 61%. Maxillofacial injuries were more frequently identified upon admission with 75 cases (12.3%) documented in the initial report and 22 additional cases (3.6%) added after retrospective review. In contrast, dental trauma was substantially underreported with only 12 of 50 cases (24%) identified at admission, and 38 cases (76%) detected retrospectively. These results underline a clear diagnostic gap in the identification of dental injuries during the acute trauma assessment. One likely explanation is the clinical context in which these patients are evaluated. The majority of DTMI-positive patients 82 out of 124 (66%) had concurrent traumatic injuries requiring urgent intervention. Among them, 33 patients (26.6%) had concomitant cerebral and/or spinal injuries such as intraparenchymal, subarachnoid, subdural hemorrhages or vertebral fractures. An additional 29 patients (23.4%) presented with both cerebral and/or spinal traumatic injuries and extracranial injuries, including rib fractures, intra-abdominal organ lacerations, or active bleedings. Only 20 patients (16.1%) had isolated facial trauma. This shows a high association of DTMI with cranial or spinal trauma due to the transmission of kinetic energy through the cervical and thoracic spine into adjacent facial structures. Interestingly, 18 of the 124 patients (14.5%) with positive findings for DTMI at review time, had no traumatic findings on the admission imaging reports, therefore we can suppose the radiologist likely failed to identify them because the absence of visible trauma leads to less attentive image review. CT is the modality of choice in trauma imaging; however, underreporting of associated injuries such as DTMI remains common. As discussed by Piccolo et al., the prioritization of life-threatening findings in polytrauma patients often leads to the neglect of coexisting craniofacial injuries, which may sometimes be identified only retrospectively. Their findings support the need for thorough multiplanar analysis and structured reporting to improve early detection of such injuries [16].

To our knowledge, this is one of the first studies to systematically assess both dental and maxillofacial injuries on trauma CT in a high-energy polytrauma setting. Previous works such as Meyer et al. (2022) have focused primarily on dental trauma detection alone reporting that dentoalveolar injuries were identified in 78 of 611 cases (12.8%) of all high-energy polytrauma cases, with only 9 of 78 (11.8%) of those sufficiently described in the original radiological reports [15]. Similarly, another study evaluating cranial CT scans demonstrated a substantial underreporting of dental trauma, with only 11% of relevant findings accurately recorded [17]. Notably, dental injuries were more likely to be reported when associated with concomitant bone fractures or paranasal sinus pathology [18].

When stratified by time of admission, 39 of the 81 (48%) of patients evaluated during daytime hours had additional findings detected during retrospective review, compared to 24 of the 43 (55%) of those evaluated during night shifts. Although slightly higher at night, this difference was not statistically significant (χ² = 0.654, *p* > 0.05), suggesting consistent diagnostic performance across shifts.

Our data also showed that men suffered significantly more injuries than women, with a ratio of 1,79:1, while patients were found to be equally distributed among all age groups [19–22]. These findings align with the statistical trends documented in existing literature. The number, type, and severity of injuries per patient varied according to age and cause of injury. Road traffic accidents emerged as the primary cause of injury [13], followed by high-energy trauma of unknown dynamics; additional causes included falls from heights, accidental falls to the ground, and physical assault confirming that maxillofacial trauma is commonly associated with high-energy impact injuries [1, 15]The most frequent injuries were UCMP’s fractures, mostly involving the nose, while the most misinterpreted finding was LAMP’s fractures where involvement was unreported in 5/20 cases (25%). The number of injuries per patient ranged from 1.1 to 2.0, although this variation could be influenced by the injuries recorded, the classification system used, and the type of study conducted. Furthermore, more severe injuries were reported in older patients. Major predisposing factors for maxillofacial trauma that may influence the severity of injury include excessive maxillary overjet and heterogeneous patterns of malocclusion or bone malformations.

This study has several limitations. Its retrospective design carries inherent biases related to data availability, variability in imaging protocols and reporting, and the emergency setting in which imaging was performed. The potential inclusion of pre-existing dental trauma represents an additional source of bias, as CT does not allow reliable temporal differentiation between acute and chronic injuries. The true incidence of dental trauma may also be underestimated, as subtle findings may have been overlooked during the CT interpretation due to the limited resolution of standard trauma CT protocols. Furthermore, the single-center design, limited sample size, may reduce the generalizability of the results and may have overlooked seasonal variations in the incidence of trauma. Finally, the lack of information on ambulance triage protocols introduces a potential selection bias in the patient population referred to our emergency department.

Despite these limitations, our findings highlight the high incidence and frequent underreporting of DTMI in initial CT assessments. Similar underreporting trends have been documented in previous studies [15], emphasizing the need for improved diagnostic protocols. Several factors contribute to this underreporting, including the primary focus on life-threatening injuries, the complex anatomical assessment required for dental structures, and limited emphasis on maxillofacial trauma in standard radiology training. Given these findings, it is imperative to implement strategies to enhance the detection of DTMI. These may include dedicated maxillofacial imaging protocols, increased awareness among radiologists, and multidisciplinary collaboration with oral and maxillofacial surgeons. Moreover, the integration of artificial intelligence tools for automated detection of DTMI may further improve diagnostic accuracy. This study complies with the guidelines of the International Association of Dental Traumatology (IADT) and considers its recommendations in the evaluation of dental and maxillofacial trauma [23].

## Conclusion

This study confirms a significant underestimation of DTMI in the acute CT evaluation of polytrauma patients. The prioritization of life-threatening injuries (like hemorrhages, thoracic or abdominal injuries) often leads to the oversight of less immediately critical, but clinically relevant, findings involving the maxillofacial and/or dental trauma.

To address this gap, future efforts should focus on optimizing imaging workflows, developing structured reporting checklists, and promoting interdisciplinary collaboration. These strategies are essential to improve diagnostic accuracy, guide appropriate treatment planning, and ensure both better patient outcomes and medicolegal protection for healthcare providers.

## Data Availability

The data that support the findings of this study are not publicly available due to privacy and ethical restrictions. However, data are available from the corresponding author upon reasonable request.

## Abbreviations

DTMI: Dental Trauma and Maxillofacial Injuries
CT: Computed Tomography
TCTB: Total–Body Computed Tomography
IADT: International Association of Dental Traumatology
AO: CMF–Arbeitsgemeinschaft für Osteosynthesefragen–CranioMaxilloFacial
CMF: CranioMaxilloFacial
SPSS: Statistical Package for the Social Sciences
DF: Degrees of Freedom
DR: Detection Rate
OR: Odds Ratio
HU: Hounsfield Units
PACS: Picture Archiving and Communication System
DS: Dual Source
MPR: Multiplanar Reformatting
MIP: Maximum Intensity Projection
TBI: Traumatic Brain Injury
LCMP: Lower Central Midface Partition
ICMP: Intermediate Central Midface Partition
UCMP: Upper Central Midface Partition
OMP: Orbital Midface Partition
LAMP: Lateral Midface Partition
PNP: Pyramid of Nose Partition
